# Real-world outcomes of pembrolizumab in advanced anal cancer: a nationwide Danish anal Cancer Group report

**DOI:** 10.2340/1651-226X.2026.44690

**Published:** 2026-05-11

**Authors:** Karen-Lise Garm Spindler, Christian Andreas Hvid, Lars Ulrik Fokdal, Karen Lycke Wind, Anne Vittrup Jakobsen, Rikke Løvendahl Eefsen, Eva Serup-Hansen

**Affiliations:** a Department of Oncology, Aarhus University Hospital, Aarhus, Denmark; b Department of Clinical Medicine, Aarhus University, Aarhus, Denmark; c Department of Oncology, Vejle Hospital, Vejle, Denmark; d Department of Oncology, Copenhagen University Hospital – Herlev and Gentofte, Copenhagen, Denmark

**Keywords:** Anal cancer, squamous cell carcinoma, immune checkpoint inhibitors, antibodies monoclonal humanised

## Abstract

**Background and purpose:**

Squamous cell carcinoma of the anal canal (SCCA) is a rare malignancy with limited treatment options for advanced or metastatic disease. Immune checkpoint inhibitors (ICIs) have shown durable responses in clinical trials, but evidence derives from small and highly selected populations. This study investigates the effectiveness and tolerability of pembrolizumab in an unselected real-world cohort.

**Patient/material and methods:**

This retrospective, multicenter cohort study evaluated the real-world efficacy, durability, and safety of pembrolizumab in Danish patients with advanced or metastatic SCCA treated between September 2018 and September 2023. A total of 37 patients, who received at least one dose of pembrolizumab for non-resectable, recurrent or metastatic disease, were included (89% ≥ 2^nd^ line). Median age was 64 years, the majority were female (64.9%), and most tumours were human papillomavirus (HPV) (p16) positive (73.0%).

**Results:**

The objective response rate (ORR) was 13.5%, with two complete and three partial responses. The clinical benefit rate (CBR) was 48.6%, and two patients had durable responses exceeding 24 months. Median progression-free survival (PFS) and overall survival (OS) were 4.0 months 95% confidence interval (CI) (2.6–5.5) and 12.1 months 95% CI (7.6–15.2), respectively. Patients with good performance status and HPV-positive disease had significantly improved survival outcomes. Treatment was well tolerated, and no treatment-related deaths were reported.

**Interpretation:**

In this real-world cohort, pembrolizumab demonstrated durable responses in a subset of patients with advanced SCCA and an acceptable safety profile. Outcomes were comparable to clinical trial data, indicating modest activity. Findings support using pembrolizumab as a treatment option in selected patients, but further evidence is needed to refine its role.

## Introduction

Squamous cell carcinoma of the anus (SCCA) is a rare malignancy, accounting for approximately 2–4% of all gastrointestinal cancers [1]. In Denmark, between 150 and 200 new cases of SCCA are diagnosed each year. Although curative-intent chemoradiotherapy is effective for localised disease, 10% of patients present with or eventually develop non-resectable locally recurrent or metastatic disease, for which the prognosis remains poor [2]. The median overall survival (OS) in this setting ranges from 12 to 22 months, depending on patient selection and line of therapy, with very limited data guiding optimal management [2, 3]. Systemic treatment for advanced SCCA is not standardised, and recommendations have historically been based on small, non-randomised studies. Platinum-based doublet chemotherapy, such as carboplatin and paclitaxel, has been adopted as the most common first-line palliative regimen. The InterAAct trial, a randomised phase II study, compared carboplatin/paclitaxel with cisplatin/5-FU and demonstrated similar efficacy between the two regimens, but a more favourable toxicity profile with carboplatin/paclitaxel, and improved survival (12.3 months vs. 20 months), which is now generally preferred in clinical practice [4]. Despite the availability of chemotherapy, outcomes in this population are suboptimal, and many patients are elderly or frail, limiting tolerance to aggressive regimens. As such, there is a pressing need for alternative and well-tolerated palliative options, especially those offering the possibility of durable disease control with manageable toxicity.

The SCCA is a biologically distinct entity from other gastrointestinal cancers. Most tumours are driven by persistent infection with high-risk human papillomavirus (HPV), which leads to expression of viral oncoproteins such as E6 and E7 [5, 6]. These foreign antigens can increase tumour immunogenicity and present an opportunity for immune recognition and attack. Moreover, SCCA shares histological and molecular features with squamous cell carcinomas of other sites (e.g. head and neck, cervix), where immune checkpoint inhibitors (ICIs) have shown substantial clinical benefit. SCCA tumours often exhibit PD-L1 expression, high levels of tumour-infiltrating lymphocytes (TILs), and other markers of immune activation, providing a strong biological rationale for using immune checkpoint blockade [7].

Clinical trials evaluating ICIs as monotherapy in advanced SCCA have demonstrated modest response rates but durable responses in a subset of patients. In the phase Ib KEYNOTE-028 and phase II KEYNOTE-158 studies, pembrolizumab achieved objective response rates (ORR) of 17% and 11%, respectively, with some long-lasting responses [8, 9]. Similarly, nivolumab demonstrated an ORR of 24% in heavily pretreated patients in the NCI9673 trial [10]. The phase II POD1UM-202 study evaluated retifanlimab in previously treated SCCA, showing improved ORR and disease control [11]. Recently, the phase III POD1UM-303 trial demonstrated a clinical benefit of adding retifanlimab to first-line carboplatin-paclitaxel with manageable toxicity [12]. Ongoing studies are also investigating combinations with anti-CTLA-4 agents, chemotherapy, or radiotherapy to enhance the immune response or modulate the tumour microenvironment [13].

Despite these promising data, most trial populations are highly selected, often excluding patients with poor performance status and multiple comorbidities. To address this gap of knowledge, real-world data are urgently needed to inform the generalisability and utility of immunotherapy in unselected patient populations and routine clinical settings. In the absence of predictive biomarkers and randomised phase III data, real-world evidence is essential to understand how immunotherapy performs outside of trial constraints. In clinical practice, oncologists must balance potential benefits with the risk of immune-related toxicities, especially in patients with limited treatment options or impaired quality of life.

This retrospective Danish multicentre study aims to contribute real-life data on efficacy, durability, and tolerability of pembrolizumab monotherapy in patients with advanced/metastatic SCCA treated across three oncology centers. The sample size is generally small due to the rarity of the disease. However, the novelty of capturing outcomes in an unselected national cohort, addresses a critical gap in current evidence and provides insight into treatment decisions in routine oncology practice.

## Patients/material and methods

We conducted a retrospective, multicenter cohort study including all patients in Denmark with histologically confirmed advanced or metastatic SCCA who received pembrolizumab monotherapy outside of clinical trials. The study is reported in accordance with the STROBE (Strengthening the Reporting of Observational Studies in Epidemiology) guidelines [14]. Patients were identified from three major oncology departments: Aarhus University Hospital, Copenhagen University Hospital – Herlev and Gentofte, and Vejle Hospital. The inclusion period spanned from September 2018 to September 2023. Eligible patients were all who had received at least one dose of pembrolizumab for non-resectable, recurrent or metastatic disease in a palliative setting during the period. Patients enrolled in interventional clinical trials were excluded.

## Data collection

Clinical data were retrospectively extracted from electronic medical records. Variables included demographics, ECOG performance status, human immunodeficiency virus (HIV) and HPV/p16 status (if available), prior lines of systemic therapy, pembrolizumab treatment dates, response assessments, adverse events, and survival outcomes.

Radiologic response was assessed according to RECIST v1.1 based on routine imaging schedules, as interpreted by local radiologists. In cases where radiologic response data were unavailable or ambiguous, clinical documentation was used to determine the best overall response. Progression-free survival (PFS) was defined as the time from initiation of pembrolizumab to radiologically or clinically confirmed disease progression or death from any cause. OS was defined as the time from pembrolizumab initiation to death from any cause.

### Statistical analysis

Descriptive statistics were used to summarise baseline characteristics and treatment patterns. Categorical variables were reported as frequencies and percentages; continuous variables as medians with interquartile ranges (IQR) or ranges. Kaplan–Meier estimates were used to calculate median PFS and OS. Hazard ratios (HR) with 95% confidence intervals (CI) were calculated using Cox proportional hazards model, and the log-rank test was applied for group comparisons. ORR and clinical benefit rate (CBR) were calculated as the proportion of patients achieving complete response (CR) or partial response (PR), and CR/PR/stable disease (SD), respectively, as best overall response. Toxicity was reported according to CTCAE v5.0 based on clinical chart review. All statistical analyses were performed using NCSS Statistical Software (NCSS, LLC, Kaysville, Utah, USA), and a *p*-value < 0.05 was considered statistically significant.

## Results

A total of 37 patients with non-resectable, recurrent or metastatic SCCA treated with pembrolizumab in routine clinical practice across Denmark were included in the study. Baseline patient characteristics are summarised in Table 1.

**Table 1:** Baseline characteristics *n* = 37.

Characteristic	*n* (%)
Age in years, median (range)	64 (40–81)
< 64 years	20 (54.1)
≥ 64 years	17 (45.9)
Sex	
Female	24 (64.9)
Male	13 (35.1)
Performance status	
0	20 (54.1)
1	11 (29.7)
2	3 (8.1)
NA	3 (8.1)
p16 status	
Positive	27 (73.0)
Negative	5 (13.5)
NA	5 (13.5)
Smoker	
Active	9 (24.3)
Former	6 (16.2)
Never	18 (48.6)
NA	4 (10.8)
HIV status	
Positive	4 (10.8)
Negative	22 (59.5)
NA	11 (29.7)
Line of therapy	
1st	4 (10.8)
2nd	28 (75.7)
3rd or later	5 (13.5)
Liver metastases	
Yes	13 (35.1)
No	24 (64.9)

HIV: human immunodeficiency virus; NA: not available.

**Figure 1:**
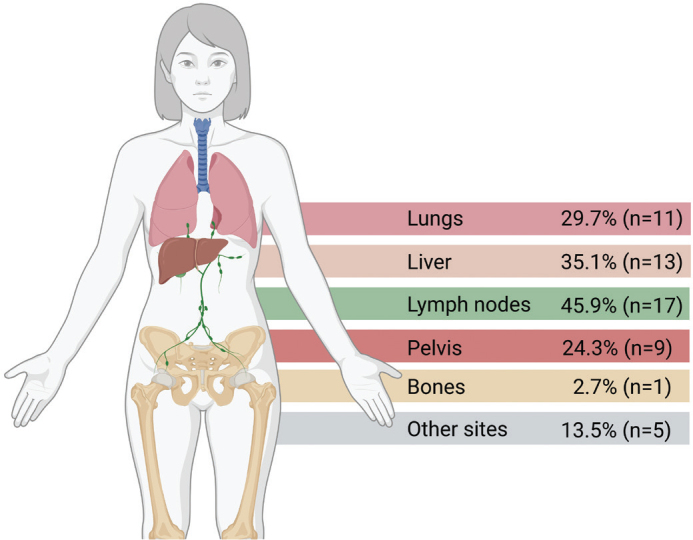
Location of metastatic lesions. Created in BioRender. Jakobsen, A. (2025) https://BioRender.com/e529iuw.

The majority were female (64.9%, *n* = 24), and most tumours were p16-positive (73.0%, *n* = 27). HIV status was not routinely tested for, but four patients were treated with known HIV positive disease. The median age was 64 years, and a total of 10 patients were 75 years or older at the time of therapy initiation (range 40–81). Only six of the treated patients had metastatic diasee at diagnosis, whereas most patients had experienced treatment failure/recurrence after localised disease. The location of metastases was mostly extra pelvic lymph node metastases (*n* = 17) as well as liver (*n* = 13) and lung metastases (*n* = 11) (Figure 1). The median follow-up time was 10.5 months (range 1.3–62.0 months).

### Therapy

Pembrolizumab was administered as first-line treatment in four patients (10.8%), second-line in 28 patients (75.7%), and third- or later-line in five patients (13.5%). The median number of pembrolizumab cycles administered was 4 (range: 1–33). The reasons for discontinuation of therapy were PD (78%), toxicity, (8%), and CR (*n* = 2). In all, 14 received another line of systemic treatment after discontinuing immunotherapy. The two patients with CR received almost 2 years of pembrolizumab (18 and 19 cycles, respectively) and afterwards patients entered follow-up.

### Efficacy

**Figure 2:**
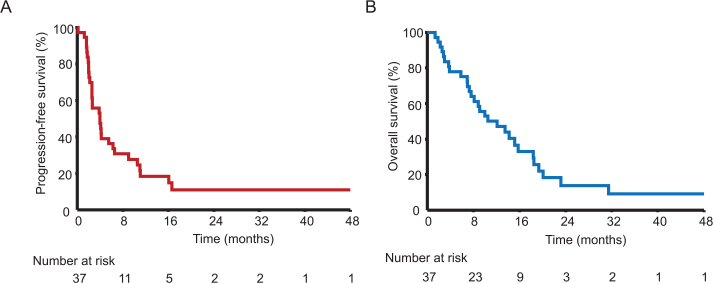
Kaplan–Meier curves for progression-free survival (PFS) and overall survival (OS) in the total cohort (A–B).

**Figure 3:**
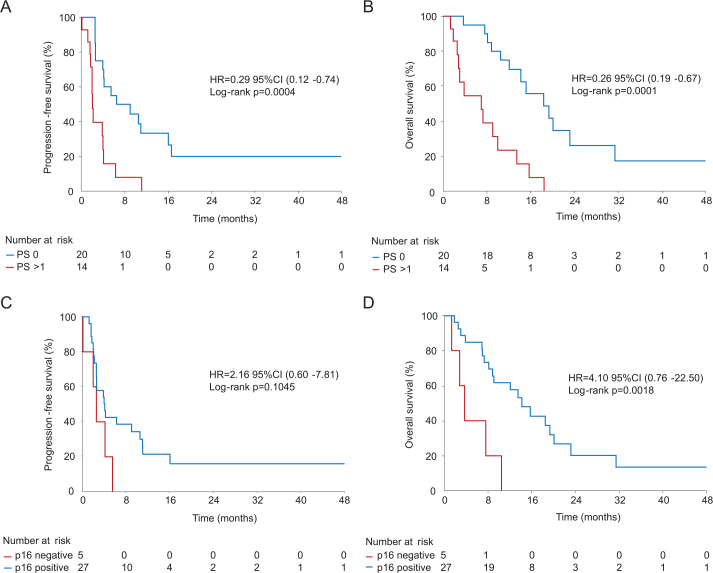
Kaplan–Meier curves for progression-free survival (PFS) and overall survival (OS) according to ECOG performance status (PS) (A–B) and HPV (p16) status (C–D). Presented with hazard ratios (HR) and 95% confidence intervals (CI), group comparison with log-rank test.

The ORR was 13.5% (*n* = 5), including two confirmed complete responses and three partial responses. CBR, defined as the proportion of patients achieving complete or partial response or stable disease as best response, was 48.6% (*n* = 18) (Table 2). Two patients experienced ongoing, durable responses exceeding 24 months without evidence of progression at last follow-up. One case with partial response had a local excision of solitary metastasis and is followed without evidence of disease. Median PFS was 4.0 months 95% CI (2.6–5.5), and median OS was 12.1 months 95% CI (7.6–15.2), as illustrated in Kaplan–Meier curves (Figure 2). Further analyses of survival according to treatment line, p16 status, and age are presented in Table 3 and Figure 3. The median OS was significantly shorter in patients with poor performance status (> 0), and HPV/p16 negative disease. The Median OS was 18.4 months 95% CI (12.1–20.1) in patients with PS 0 compared to 7.0 months 95% CI (2.8–9.1) in the remaining group, (HR 0.26 95% CI (0.19–0.67), *p* = 0.0001). Patients with HPV/p16 negative disease experienced a shorter median OS than those with HPV-dependent disease, 3.7 months 95% CI (1.3–10.5) and 14.2 months 95% CI (8.8–19.4), (HR 4.10 95% CI(0.76–22.50), *p* = 0.0018). All patients with partial and durable responses were in PS group 0 and with HPV-dependent disease.

**Table 2:** Efficacy outcomes.

Outcome	Value
Objective response rate (ORR)	13.5%
Clinical benefit rate (CBR)	48.6%
Complete responses (CR)	5.4% (*n* = 2)
Durable responses > 24 months	5.4% (*n* = 2)
Median progression free survival (PFS) in months (95% CI)	4.0 (2.6–5.5)
Median overall survival (OS) in months (95% CI)	12.1 (7.6–15.2)

CI. confidence interval.

**Table 3:** Univariate log-rank tests of overall survival (OS) according to pre-treatment parameters.

Variable	HR	95% CI	*p*-value
Age	0.72	0.33–1.55	0.37
Gender	0.84	0.39–1.81	0.64
Performance status (> 0)	0.26	0.19–0.67	0.0001
HPV (p16) status	4.10	0.76–22.50	0.0018
Treatment line	0.72	0.25–2.07	0.53
Liver metastases	0.53	0.23–1.19	0.08

HR: hazard ratio; CI: confidence interval.

### Safety

Pembrolizumab was generally well tolerated. Most adverse events were grade 1–2. Two patients (5.4%) required hospitalisation due to grade 3 immune-related adverse events. Five patients received short time cortisol/prednisolone as part of supportive care. There were no treatment-related deaths. Two patients discontinued treatment due to toxicity during the observation period.

## Discussion

This retrospective nationwide study presents real-world data on the efficacy and safety of pembrolizumab monotherapy in patients with advanced or metastatic SCCA treated across Denmark outside of clinical trials. Our findings suggest that pembrolizumab is a viable treatment option in this rare patient population, with outcomes that are comparable to those reported in clinical trials, including durable responses in a subset of patients and an acceptable toxicity profile. An overview of studies is presented in Table 4.

**Table 4:** An overview of studies of immunotherapy in advanced SCCA.

Study / Author	Treatment	*N*	Line of therapy	ORR (%)	Median PFS (mo)	Median OS (mo)
Monotherapy						
KEYNOTE-028 (Ott et al. 2017) [8]	Pembrolizumab	24	≥ 1st	17	3.0	9.3
KEYNOTE-158 (Marabelle et al. 2022) [9]	Pembrolizumab	112	≥ 1st	11	2.0	11.9
NCI9673 (Morris et al. 2017) [10]	Nivolumab	37	> 1st	24	4.1	11.5
POD1UM-202 (Rao et al. 2022) [11]	Retifanlimab	94	≥ 1st	14	2.3	10.1
Huffman et al. 2023 [15]	Pembrolizumab	32	≥ 1st	9	2.2	13.6
Current Danish Study	Pembrolizumab	37	1st– ≥ 3rd	14	4.0	12.1
Combinations with chemotherapy						
POD1UM 303 (Rao et al. 2025) [12]	Carboplatin/Paclitaxel +/- Retifanlimab	308	1st	56/44	9.3/7.4	29.2/23.0
SCARCE (Kim et al. 2024) [16]	mDCF +/- Atezolizumab	97	1st	75/78	9.4/8.7	NR
Other combinations						
CARACAS (Lonardi et al. 2021) [17]	Avelumab +/- Cetuximab	60	> 1st	17/10	3.9/2.0	7.8/13.9
Morris et al. 2025 [18]	Atezolizumab + Bevacizumab	20	> 1st	11	4.1	11.6

ORR: objective response rate; PFS: progression-free survival; mo: months; OS: overall survival; mDCF: modified docetaxel, cisplatin, and fluorouracil; NR: not relevant; Response rates are rounded to full numbers.

The observed ORR of 13.5% in our cohort is consistent with the results from KEYNOTE-158, where the ORR was 11% but higher than the ORR of 9.4% reported in Huffman et al. Notably, the CBR of 48.6% in our study was higher than in both studies, which demonstrated CBRs of 26% and 21.9%, respectively [9, 15]. Similarly, the POD1UM-202 trial evaluating retifanlimab in previously treated advanced SCCA demonstrated an ORR of 13.8% and CBR of 48.9% closely mirroring outcomes seen with pembrolizumab [11]. The NCI9673 study of nivolumab in pretreated SCCA reported a higher ORR (24%), but in a highly selected population [10]. This study from the Danish Anal Cancer Group (DACG) includes an unselected, real-world population, encompassing elderly and comorbid patients often underrepresented in trials. Importantly, two patients in our study experienced durable clinical benefit exceeding 24 months, highlighting the potential for long-term disease control in selected individuals.

The median PFS (4.0 months) and OS (12.1 months) observed in our cohort are comparable to or modestly better than published trial data, possibly reflecting careful patient selection and follow-up in routine clinical practice. Furthermore, the median number of administered cycles (4) and the wide range (1–33) illustrate the variability in treatment duration and underline the importance of early identification of responders and non-responders to optimise resource use and avoid unnecessary toxicity.

In terms of safety, pembrolizumab was generally well tolerated, with only two patients experiencing grade 3 adverse events requiring hospitalisation and no treatment-related deaths. Importantly, nearly half of our patients were aged ≥ 64 years (up to 81 years), and pembrolizumab remained well tolerated in this elderly subgroup. This reinforces immunotherapy as a favourable option for elderly and frail or pretreated patients where the toxicity burden of chemotherapy may be unacceptable. However, the benefit of immunotherapy must be balanced with its limited efficacy in the majority of patients and the need for reliable predictive biomarkers to guide patient selection.

In SCCA mismatch-repair deficiency (dMMR)/MSI-high is very rare and tumour mutational burden is generally low, limiting their utility as predictive markers for PD-1 inhibition. PD-L1 expression is more frequent (35–60%) and may enrich for response in some cohorts, but results are variable and no single biomarker reliably identifies patients most likely to benefit. Our study demonstrated better outcomes among patients in good performance status and with HPV-positive disease, warranting further evaluation in larger datasets. International collaboration is needed to provide statistically reliable sample sizes for these purposes.

Combination regimens have been explored in the SCARCE trial on modified docetaxel, cisplatin and fluorouracil with or without atezolizumab, which showed no difference in outcome [16]. Contrastingly, the phase III POD1UM-303 trial with retifanlimab and chemotherapy, have shown encouraging improvements in response rates and meaningful clinical benefits compared to chemotherapy alone in the first line [12]. Other combination strategies beyond chemotherapy have also been explored in the CARACAS trial evaluating avelumab with cetuximab and the study by Morris et al. investigating atezolizumab with bevacizumab, both showing only modest activity in previously treated patients [17, 18]. The added toxicity of combination therapies remains a concern, particularly in the palliative setting. In this context also, real-world data such as ours must provide essential insights into treatment outcomes, tolerability, and quality of life trade-offs, especially in unselected populations.

It is suggested that the efficacy of ICIs may be more limited in later lines. In KEYNOTE-158, most patients had received ≥ 1 prior systemic therapy, and responses, though durable, were observed in a minority [9]. Similarly, in our study, durable responses occurred in patients treated as a second-line option, but in a small number of patients. This could support the hypothesis that earlier introduction of immunotherapy, before significant immunosuppression from prior lines or disease progression, may increase the likelihood of benefit. However, only four patients in our cohort received pembrolizumab as first-line therapy, limiting our ability to draw definitive conclusions about its efficacy in the frontline setting. The high response rates observed in POD1UM-303 [12], which evaluated ICIs in combination with chemotherapy in the first line, suggest a potential benefit in earlier use. Ongoing trials may further clarify the optimal sequencing of ICIs in SCCA.

The relatively low response rates for ICI monotherapy can thus be questioned. However, while combination chemo-immunotherapy in first-line settings, demonstrates encouraging high response and survival rates, single-agent PD-1 blockade could also remain an attractive option for selected patients. A CBR of approximately 50% and potential for durable response, coupled with limited toxicity, may be meaningful in frail or treatment-exhausted patients where quality of life and tolerability are key considerations. Treatment decisions in this context should therefore reflect a balanced, shared decision-making process between physician and patient. But since efficacy in the elderly remains variable, biomarkers and geriatric assessment tools would be beneficial for an improved treatment selection for the patients. Future biomarkers could imply alternative response evaluation tools such as the early response evaluation based on measurements of circulating tumour DNA in liquid biopsies, or selection based on pre-treatment marker for favourable prognosis.

Limitations of this study include the retrospective design and a small sample size. Larger sample sizes and/or prospective studies are needed to allow for combined analysis of multiple other relevant parameters, such as other metastatic sites, HIV status, patients history and comorbidities. Other limitations are a lack of centralised radiologic review or biomarker data such as PD-L1 or tumour mutational burden. Nevertheless, this is one of the few real-world cohorts reported to date in Europe and provides valuable insight into treatment outcomes with ICI in routine clinical practice.

## Data Availability

The data in this study are not publicly available due to data protection regulations. However, pseudonymised data can be made available upon reasonable request to the corresponding author, subject to necessary approvals from relevant authorities.

## References

[1] Islami F, Ferlay J, Lortet-Tieulent J, Bray F, Jemal A. International trends in anal cancer incidence rates. Int J Epidemiol. 2017;46(3):924–38. 10.1093/ije/dyw276 27789668

[2] Johnsson A, Norman D, Angenete E, Cavalli-Björkman N, Lagerbäck C, Leon O, et al. Anal cancer in Sweden 2015–2019. Implementation of guidelines, structural changes, national registry and early results. Acta Oncol. 2022;61(5):575–82. 10.1080/0284186X.2022.2048069 35274596

[3] Rao S, Guren MG, Khan K, Brown G, Renehan AG, Steigen SE, et al; ESMO Guidelines Committee. Electronic address: clinicalguidelines@esmo.org. Anal cancer: ESMO Clinical Practice Guidelines for diagnosis, treatment and follow-up☆. Ann Oncol. 2021;32(9):1087–100. 10.1016/j.annonc.2021.06.015 34175386

[4] Rao S, Sclafani F, Eng C, Adams RA, Guren MG, Sebag-Montefiore D, et al. International rare cancers initiative multicenter randomized phase ii trial of cisplatin and fluorouracil versus carboplatin and paclitaxel in advanced anal cancer: InterAAct. J Clin Oncol. 2020;38(22):2510–8. 10.1200/JCO.19.03266 32530769 PMC7406334

[5] Scheffner M, Werness BA, Huibregtse JM, Levine AJ, Howley PM. The E6 oncoprotein encoded by human papillomavirus types 16 and 18 promotes the degradation of p53. Cell. 1990;63(6):1129–36. 10.1016/0092-8674(90)90409-8 2175676

[6] Werness BA, Levine AJ, Howley PM. Association of human papillomavirus types 16 and 18 E6 proteins with p53. Science. 1990;248(4951):76–9. 10.1126/science.2157286 2157286

[7] Sclafani F, Rao S. Systemic therapies for advanced squamous cell anal cancer. Curr Oncol Rep. 2018;20(7):53. 10.1007/s11912-018-0698-6 29728940

[8] Ott PA, Piha-Paul SA, Munster P, Pishvaian MJ, van Brummelen EMJ, Cohen RB, et al. Safety and antitumor activity of the anti-PD-1 antibody pembrolizumab in patients with recurrent carcinoma of the anal canal. Ann Oncol. 2017;28(5):1036–41. 10.1093/annonc/mdx029 28453692 PMC5406758

[9] Marabelle A, Cassier PA, Fakih M, Kao S, Nielsen D, Italiano A, et al. Pembrolizumab for previously treated advanced anal squamous cell carcinoma: results from the non-randomised, multicohort, multicentre, phase 2 KEYNOTE-158 study. Lancet Gastroenterol Hepatol. 2022;7(5):446–54. 10.1016/S2468-1253(21)00382-4 35114169 PMC12012850

[10] Morris VK, Salem ME, Nimeiri H, Iqbal S, Singh P, Ciombor K, et al. Nivolumab for previously treated unresectable metastatic anal cancer (NCI9673): a multicentre, single-arm, phase 2 study. Lancet Oncol. 2017;18(4):446–53. 10.1016/S1470-2045(17)30104-3 28223062 PMC5809128

[11] Rao S, Anandappa G, Capdevila J, Dahan L, Evesque L, Kim S, et al. A phase II study of retifanlimab (INCMGA00012) in patients with squamous carcinoma of the anal canal who have progressed following platinum-based chemotherapy (POD1UM-202). ESMO Open. 2022;7(4):100529. 10.1016/j.esmoop.2022.100529 35816951 PMC9463376

[12] Rao S, Samalin-Scalzi E, Evesque L, Ben Abdelghani M, Morano F, Roy A, et al. POD1UM-303/InterAACT-2 study investigators. Retifanlimab with carboplatin and paclitaxel for locally recurrent or metastatic squamous cell carcinoma of the anal canal (POD1UM-303/InterAACT-2): a global, phase 3 randomised controlled trial. Lancet. 2025;405(10495):2144–52. 10.1016/S0140-6736(25)00631-2 40517007

[13] Dhawan N, Afzal MZ, Amin M. Immunotherapy in anal cancer. Curr Oncol. 2023;30(5):4538–50. 10.3390/curroncol30050343 37232801 PMC10217754

[14] von Elm E, Altman DG, Egger M, Pocock SJ, Gøtzsche PC, Vandenbroucke JP; STROBE Initiative. The Strengthening the Reporting of Observational Studies in Epidemiology (STROBE) statement: guidelines for reporting observational studies. Lancet. 2007;370(9596):1453–7. 10.1016/S0140-6736(07)61602-X 18064739

[15] Huffman BM, Singh H, Ali LR, Horick N, Wang SJ, Hoffman MT, et al. Biomarkers of pembrolizumab efficacy in advanced anal squamous cell carcinoma: analysis of a phase II clinical trial and a cohort of long-term responders. J Immunother Cancer. 2024;12(1):e008436. 10.1136/jitc-2023-008436. 38272561 PMC10824013

[16] Kim S, Ghiringhelli F, de la Fouchardière C, Evesque L, Smith D, Badet N, et al. Atezolizumab plus modified docetaxel, cisplatin, and fluorouracil as first-line treatment for advanced anal cancer (SCARCE C17-02 PRODIGE 60): a randomised, non-comparative, phase 2 study. Lancet Oncol. 2024;25(4):518–28. 10.1016/S1470-2045(24)00081-0 38547895

[17] Lonardi S, Prete AA, Morano F, Messina M, Formica V, Corsi DC, et al. Randomized phase II trial of avelumab alone or in combination with cetuximab for patients with previously treated, locally advanced, or metastatic squamous cell anal carcinoma: the CARACAS study. J Immunother Cancer. 2021;9(11):e002996. 10.1136/jitc-2021-002996 34815354 PMC8611452

[18] Morris VK, Liu S, Lin K, Zhu H, Prasad S, Mahvash A et al. Phase II trial of atezolizumab and bevacizumab for treatment of HPV-positive unresectable or metastatic squamous cell carcinoma of the anal canal. Clin Cancer Res. 2025;31(9):1657–66. 10.1158/1078-0432.CCR-24-1512 40019482 PMC12010964

